# Machine learning uncovers previously undetectable genetic deletions in cancer panel sequencing

**DOI:** 10.1038/s41698-026-01756-9

**Published:** 2026-10-16

**Authors:** Irina A. Kerle, Oleksandr Husak, Thomas Gross, Andreas Hartig, Rebecca Prause, Daniela Richter, Sascha Brückmann, Maximilian Werner, Anja Kögler, Dorothea Hanf, Lino Möhrmann, Doreen William, Karsten Wendt, Nicole Pfarr, Carolin Mogler, Daniela E. Aust, Evelin Schröck, Christoph Heining, Hanno Glimm

**Affiliations:** 1 Department for Translational Medical Oncology, National Center for Tumor Diseases Dresden (NCT/UCC), a partnership between German Cancer Research Center (DKFZ), Faculty of Medicine and University Hospital Carl Gustav Carus, TUD Dresden University of Technology and Helmholtz-Zentrum Dresden - Rossendorf (HZDR), Dresden, Germany; 2 Translational Medical Oncology, Faculty of Medicine and University Hospital Carl Gustav Carus, TUD Dresden University of Technology, Dresden, Germany; 3 German Cancer Consortium (DKTK), partner site Dresden, a partnership between DKFZ and University Hospital Carl Gustav Carus Dresden, Dresden, Germany; 4 Chair of Software Technology, Faculty of Computer Science, Technical University Dresden, Dresden, Germany; 5 Core Unit for Molecular Tumor Diagnostics (CMTD), National Center for Tumor Diseases Dresden (NCT/UCC), a partnership between German Cancer Research Center (DKFZ), Faculty of Medicine and University Hospital Carl Gustav Carus, TUD Dresden University of Technology and Helmholtz-Zentrum Dresden-Rossendorf (HZDR), Dresden, Germany; 6 Institute of Pathology, Carl Gustav Carus University Hospital, Dresden, Germany; 7 Department of Internal Medicine I, University Hospital Carl Gustav Carus, TUD Dresden University of Technology, Dresden, Germany; 8 Computational Health Informatics Program, Boston Children’s Hospital, Harvard Medical School, Boston, USA; 9 Internal Medicine C, Hematology, Oncology, Stem Cell Transplantation and Palliative Care, University Medicine Greifswald, Greifswald, Germany; 10 Institute for Clinical Genetics, University Hospital Carl Gustav Carus at TU Dresden, Dresden, Germany; 11 ERN GEN-TURIS, Hereditary Cancer Syndrome Center Dresden, Dresden, Germany; 12 National Center for Tumor Diseases (NCT), NCT/UCC Dresden, a partnership between DKFZ, Faculty of Medicine and University Hospital Carl Gustav Carus, TUD Dresden University of Technology, and Helmholtz-Zentrum Dresden-Rossendorf (HZDR), Germany; German Cancer Consortium (DKTK), partner site: Dresden, and German Cancer Research Center (DKFZ), Heidelberg, Germany; 13 Institute of Pathology, School of Medicine and Health, Technical University Munich, Munich, Germany

**Keywords:** Biomarkers, Cancer, Oncology

## Abstract

Missed genetic deletions can prevent cancer patients from receiving effective targeted therapy. Standard pipelines, such as TSO500, use bin count averaging for copy number variation detection but often overlook focal events. To address this, we developed a machine learning (ML) pipeline for deletion detection and zygosity evaluation within the MASTERsg precision oncology program. We focused on the tumor suppressor gene PTEN, whose loss confers sensitivity to mTOR/Akt inhibition. An Extreme Gradient Boosting model trained on PTEN-deleted and wild-type tumor samples identified focal deletions missed by the TSO500 pipeline and CNVkit that were associated with concordant PTEN protein loss by immunohistochemistry. Importantly, the model detected PTEN deletions in 33/204 profiled samples that were classified as PTEN wild-type by TSO500; 75% (15/20) of stainable cases showed absent or reduced PTEN protein expression. These findings demonstrate the potential of ML to enhance panel sequencing, uncovering therapeutically relevant alterations missed by established diagnostic methods.

## Introduction

Molecularly guided therapies play an increasingly important role in treating advanced cancers, especially when standard options are exhausted^1^. Panel sequencing is a common clinical practice in cancers with known recurrent driver alterations, such as melanoma and non-small cell lung, colorectal, breast, ovarian, or prostate cancer^2^. Conversely, patients with rare or common cancers without known driver alterations are often excluded from explorative molecular diagnostics. The subgenomic Molecularly Aided Stratification for Tumor Eradication Program (MASTERsg) at the National Center for Tumor Diseases/University Cancer Center (NCT/UCC) in Dresden, Germany, is a prospective clinical panel sequencing program using the TruSight Oncology 500 DNA (TSO500) sequencing assay^3^, covering 523 genes for single nucleotide variations (SNVs) and small insertions or deletions (indels), and a complementary germline assessment of 101 genes involved in tumorigenesis. The program aims to identify therapeutically relevant alterations in adult patients with relapsed and progressive cancers needing systemic therapies beyond established lines. In addition, the TSO500 pipeline assesses more than 500 copy number variations (CNVs) by partitioning the CNV caller’s raw data into discrete, gene-specific segments called “bins” consisting of 50–250 base pairs (bp). For each bin, a read count is tallied, and gene-level copy number estimates are derived by averaging the normalized read counts across all bins spanning the gene. However, by averaging read counts across broader regions, this approach may mask intragenic events such as focal amplifications or deletions that do not reach the caller’s fold-change thresholds^4^. This presents a major unmet clinical need, as especially undetected focal deletions may cause omission of targetable alterations, preventing access to effective therapies for cancer patients with limited options.

Although numerous computational tools have been evaluated for CNV detection across various sequencing types^5^, no published independent deletion calling algorithms are based on the TSO500 panel sequencing output.

To address this critical clinical challenge, we enhanced the CNV-calling capabilities of the TSO500 pipeline by incorporating focal deletions, thereby increasing the sensitivity and specificity of deletion detection. We used samples with validated deletions of Phosphatase and Tensin homolog (PTEN) as clinically relevant proof-of-concept. PTEN is a tumor suppressor gene that is frequently inactivated by deletions or mutations in various malignancies. Its loss has been linked to therapeutic vulnerabilities, including sensitivity to mTOR and Akt inhibitors, PARP inhibition, and resistance to immune checkpoint blockades^6^.

Machine learning (ML) has shown the potential to enhance the precision of cancer diagnosis and personalized therapy^7^. We chose to employ a gradient boosting model, a powerful ML technique that trains a series of simple models with each one correcting the prediction errors of the previous one to achieve a high accuracy^8^. Here, we used a data-driven approach to develop two models to predict deletions of PTEN based on interim data from the standard TSO500 pipeline and sample quality information. Our goal was to develop a robust and scalable software solution applicable at the single-patient level for reporting in a molecular tumor board.

By enhancing the deletion calling of PTEN in the TSO500 assay, this study aimed to find new generalizable strategies to improve deletion detection, with the potential to extend this method to other clinically relevant genes in panel sequencing.

## Results

### Benchmark performance of deletion detection

**Fig. 1:**
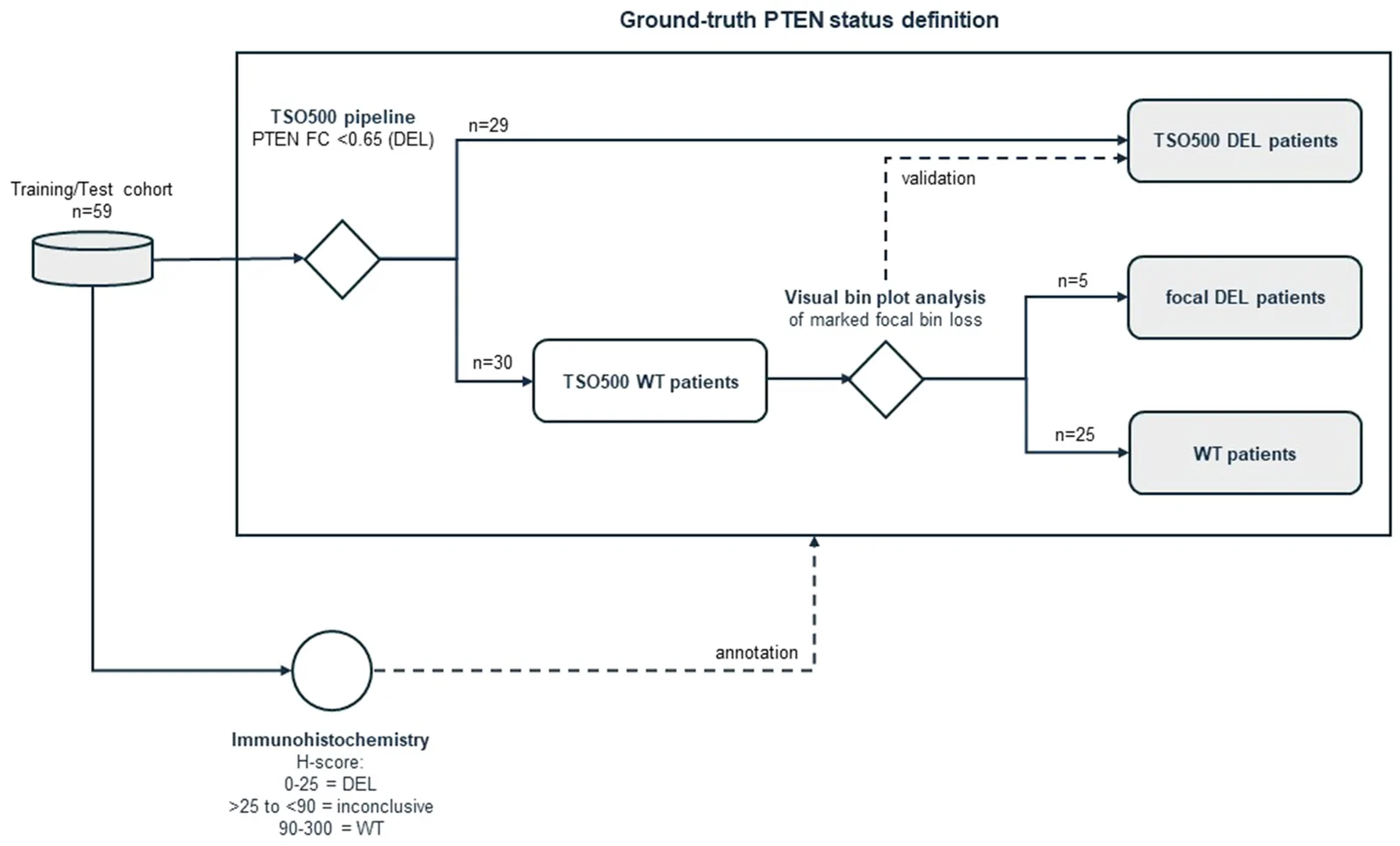
Ground-truth definition of PTEN status in the training/test cohort. Flow chart illustrating the assignment of PTEN status in the 59 patient samples from 15 solid tumor entities used for model development. TSO500 v2.2.0.2 pipeline analysis classified 29 samples as PTEN-deleted (DEL) based on a PTEN-specific fold-change (FC) threshold of <0.65, while 30 samples were classified as PTEN wild-type (WT). Visual inspection of normalized bin-level coverage data confirmed PTEN deletion status of the 29 TSO500 DEL samples and the wild-type status of 25 samples, while identifying focal PTEN deletions in five samples not detected by the TSO500 pipeline. Immunohistochemistry (IHC) was performed on all 59 samples as an orthogonal assessment of PTEN protein expression, with H-scores of 0–25, >25 to <90, and 90–300 classified as PTEN protein loss, inconclusive expression, and retained expression, respectively.

Based on our experience, the TSO500 pipeline misses focal genomic deletions with potentially significant clinical consequences. To test this hypothesis and establish a training/test cohort for subsequent ML model development, we retrospectively evaluated PTEN copy-number status using TSO500 version (v) 2.2.0.2 pipeline output and visual inspection of normalized bin-level coverage data. We assembled a training/test cohort of 59 patient samples from 15 solid tumor entities comprising (i) 29 samples classified as PTEN-deleted by the TSO500 pipeline, (ii) 5 additional samples with focal PTEN deletions not called by TSO500 but identified by visual bin plot analysis by the program’s bioinformaticians, and (iii) 25 samples classified as PTEN wild-type (WT) controls (Fig. 1, Table 1, and Supplementary Table 1).

**Table 1:** Cohort description

Cohort name	Train/Test WT	Train/Test del	Train/Test add. focal del	Train/Test (total)	Validation A	Validation B	Validation C	Validation B + C	ext. Validation
*n*	25	29	5	**59**	**10**	33	31	**64**	**61**
excluded	0	1	0	**1**	**0**	4	0	**4**	**0**
Tumor Cell Content (TCC)									
median	75	85	80	**80**	**70**	80	70	**77.50**	**60**
Fold Change (FC)									
median	0.957	0.558	0.767		**0.580**	0.750	0.991		**0.893**
min	0.650	0.046	0.658		**0.150**	0.650	0.842		**0.088**
max	1.096	0.636	1.051		**1.047**	0.970	1.261		**1.359**
H-score									
available	25	28	5	**58**	**8**	20	17	**37**	**50**
not available	0	0	0	**0**	**2**	9	14	**23**	**11**
median	165	20	0		**0**	10	80		**90**
min	0	0	0		**0**	0	0		**0**
max	300	300	35		**10**	270	280		**300**
deleted (0 ≤ 25)	1	15	4	**20**	**8**	11	1	**12**	**18**
grey area (>25−<90)	4	7	1	**12**	**0**	4	8	**12**	**6**
WT (≥90)	20	6	0	**26**	**0**	5	8	**13**	**26**
Zygozity evaluation									
homozygous del	0	13	2	**15**	**2**	0	0	**0**	**13**
heterozygous del	2	15	3	**20**	**8**	7	0	**7**	**3**
WT	23	0	0	**23**	**0**	26	31	**57**	**45**

Training/test cohort comprising retrospective samples called as PTEN deleted (del) or wild-type (WT) by the TSO500 pipeline plus five additional focal deletions identified by visual bin plot analysis. Validation cohort A comprising prospective samples identified as deleted by the TSO500 v2.2.0.2 pipeline or focal deletions detected by visual bin plot analysis and confirmed by immunohistochemistry (IHC) used to test the recall of the machine learning (ML) models. Validation cohort B + C comprising samples identified as potentially PTEN deleted by Model 1 but not by TSO500 v2.2.0.2 (validation cohort B) in retrospective samples not part of the training/test cohort, including randomly selected, entity matched samples with no detection of a PTEN deletion by the TSO500 v2.2.0.2 pipeline, visual bin plot analysis or the ML models (cohort C). External validation cohort comprising retrospective samples randomly selected without preselection based on PTEN status. Tumor cell content (TCC) statistics were based on pathological assessment. Fold-change (FC) statistics were calculated using TSO500 pipeline v2.2.0.2 for the training/test cohort and validation cohorts A–C, and using TSO500 pipeline v2.6.0.2 for the external validation cohort. H-score statistics were based on IHC staining from the samples available for staining. Zygosity evaluations were based on visual bin plot analysis by bioinformaticians.

The bold values indicate the sums of the individual patient cohorts.

In the PTEN WT cohort, the median PTEN fold-change (FC) was close to 1, with a value of 0.96 (range, 0.65–1.10), and a median H-score of 165 (range, 0–300). Bioinformaticians’ zygosity evaluation showed heterozygous deletion in 2 out of 25 cases and WT in the remaining 23. One outlier (patient 42) exhibited discordant results (PTEN FC 0.93, H-score 0), likely reflecting post-transcriptional mechanisms. The sample was retained in the training set.

The 29 TSO500-identified PTEN-deleted samples had a median FC of 0.56 (range, 0.05–0.64), which was below the PTEN-specific threshold (FC 0.65), and a median H-score of 20 (range, 0–300). Zygosity evaluation distinguished between 14 homozygous and 15 heterozygous deletions. Three patients had focal deletions classified by both visual bin plot analysis and TSO500 pipeline. One sample (patient 2) was excluded from model training due to an unexplained inconsistency between sequencing and staining (PTEN FC 0.10, H-score 220).

**Fig. 2:**
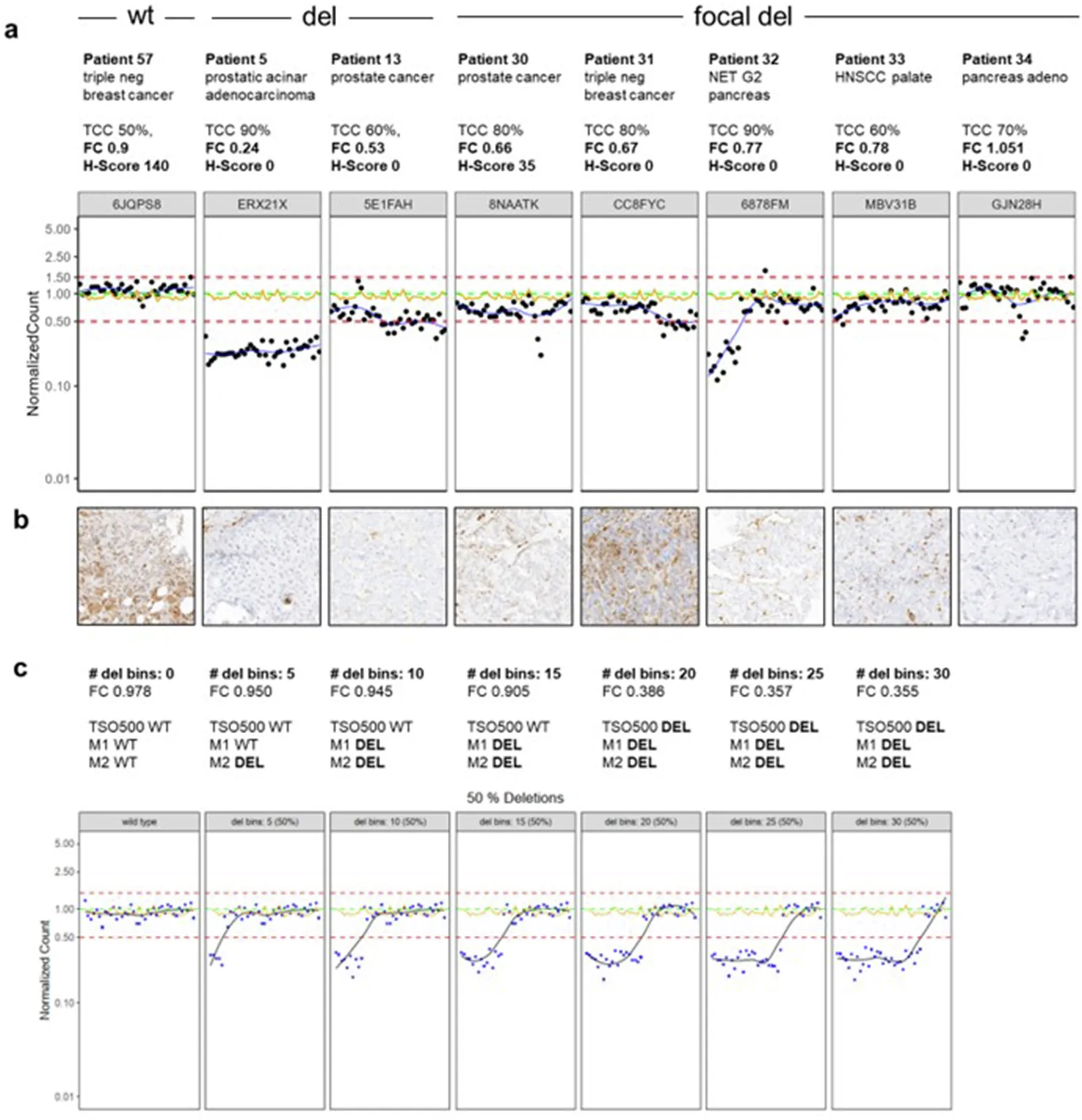
TSO500 v2.2.0.2 PTEN normalized bin plot analyses. **a** PTEN normalized bin plots of eight exemplary patient samples (left to right): one wild-type (WT), two PTEN-deletions (del) detected by the TSO500 pipeline, and five focal deletions missed by TSO500 due to average fold-change (FC) values exceeding the PTEN-specific threshold of 0.65. X-axis: PTEN bin distribution across gene length; Y-axis: FC. Dashed green line: FC 1 (WT); dotted red lines: FC 1.5 (in-house amplification threshold) and FC 0.5 (in-house deletion threshold); orange line: in-house reference; blue line and black dots: patient-specific bin plot topography for visual bin plot analysis. H-score results confirm PTEN protein presence for WT (≥90) and shortage or absence for deletions (0–25). **b** Corresponding immunhistochemical (IHC) staining images of PTEN protein expression. **c** Set of generated in silico samples with increasing number of deleted bins (50% deletion), called by either the TSO500 pipeline, machine learning (ML) Model 1 (M1) and Model 2 (M2) as PTEN deleted (DEL). # del bins: number of deleted bins; for plot description see (**a**).

Five additional cases with focal deletions were missed by the TSO500 pipeline because their FCs exceeded the PTEN-specific threshold of 0.65 but were detected by visual bin plot analysis, had a median PTEN FC of 0.77 (range, 0.66–1.05). PTEN loss was confirmed by an H-score of 0 in four cases and was suggestive (H-score 35) in one case (Fig. 2a, b).

More detailed patient data, including full zygosity evaluations and tumor cell contents (TCCs), are shown in Table 1 and Supplementary Table 1.

To illustrate the limit of detection of the TSO500 v2.2.0.2 pipeline, a set of in silico samples with increasing numbers of bins with reduced read counts was generated at 50% (Fig. 2c) and 100% reduction per bin (Supplementary Fig. 1). Deletion detection by the TSO500 pipeline required a minimum of 20 reduced bins out of the 39 PTEN bins at both reduction levels (Fig. 2c and Supplementary Fig. 1).

### Development and performance testing of PTEN deletion calling using machine learning approaches

**Fig. 3:**
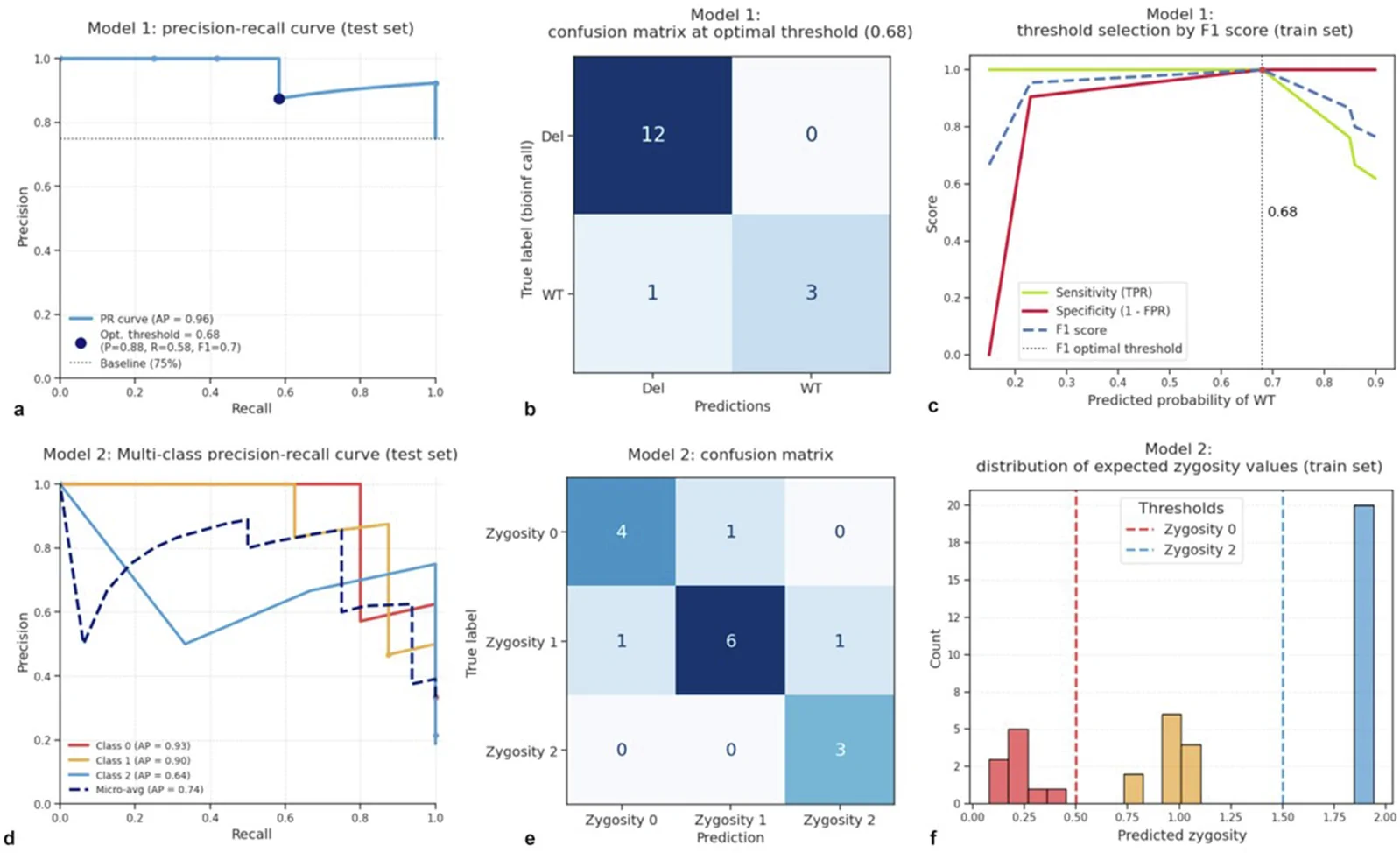
Model performance on training/test cohort. **a** Precision-recall curve of Model 1 on the independent test set. PTEN deletion is defined as the positive class (baseline = prevalence = 0.75), yielding an average precision (AP) of 0.96. At the optimal decision threshold of 0.68, determined on the training set (see **c**), Model 1 achieved a precision of 0.92, a sensitivity of 1.00, and an F1 score of 0.96 on the test set. **b** Test set classification performance at the optimal decision threshold of 0.68. All 12 PTEN deletions, including five focal events, were correctly identified. One of four PTEN wild-type (WT) samples was incorrectly classified as deleted (specificity = 0.75). **c** Threshold optimization for Model 1 on the training set. Sensitivity (yellow), specificity (red), and F1 score (blue dashed line) are plotted as functions of the predicted WT probability. The vertical dotted line indicates the threshold that maximizes the F1 score and was applied to both the test set (A, B) and validation cohort analyses. **d** Model 2 precision-recall curves for zygosity prediction (0 = homozygous deletion, 1 = heterozygous deletion, 2 = WT). AP was highest for homozygous (0.93) and heterozygous deletions (0.90), followed by WT classification (0.64). **e** Confusion matrix for Model 2, showing model predictions (x-axis) vs. bioinformaticians’ assessment based on copy number profile and tumor cell content (y-axis). Overall multiclass performance yielded a mean AP of 0.81, with 13/16 samples correctly classified, and three misclassifications occurring for both homozygous and heterozygous deletions. **f** Distribution of expected zygosity values produced by Model 2 for the train set. Dashed red (0.5) and blue (1.5) lines indicate default thresholds to discretize the continuous output into the three ordinal classes depicted in (**d**, **e**).

Two models were trained for PTEN deletion detection: Model 1 distinguishes between PTEN-deleted and WT samples by predicting the probability of a sample retaining PTEN WT, while Model 2 provides a multiclass zygosity classification (0 = homozygous deletion, i.e., both alleles deleted; 1 = heterozygous deletion, i.e., one allele deleted; 2 = WT; see “Methods” section). Model performance was evaluated using the average precision (AP) as the primary threshold-independent metric, which summarizes the precision-recall curve and is particularly informative for the positive (deleted) class. Additionally, the F1 score at the selected decision threshold was used to assess predictive performance at the chosen decision threshold (see “Methods” section). Model 1 achieved good performance during training in distinguishing PTEN-deleted samples from WT samples in the test set, achieving an AP of 0.96 (Fig. 3a, b) and an F1 score of 0.96 (Supplementary Table 2). At the optimal decision threshold of 0.68 (Fig. 3c), Model 1 correctly identified 12 PTEN deletions. Notably, these included the five focal deletions missed by the TSO500 v2.2.0.2 pipeline. The specificity was 0.75, indicating that 3 of 4 WT samples were correctly identified (Fig. 3b and Supplementary Table 2).

Model 2 extends beyond binary classification to predict PTEN zygosity status (0, 1, or 2 copies). To assess how each zygosity class contributed to the overall prediction of Model 2, we analyzed multiclass precision-recall curves. This series of graphs provides insight into class-specific performance, individually showing the performance of the model for homozygous, heterozygous, and WT samples. Multiclass precision-recall curves showed strong but variable performance: AP scores were 0.93, 0.90, and 0.64, and F1 scores were 0.80, 0.80, and 0.86 for homozygous (0), heterozygous deletion (1), and WT (2) classes, respectively (Fig. 3d and Supplementary Table 2). Model 2 correctly classified four of the five homozygous deletions, six of the eight heterozygous deletions, and all three WT samples (Fig. 3e). The model did not show confusion between zygosity states 0 and 2 (Fig. 3e). The distribution of predicted zygosity values showed clear separation, with default thresholds delineating the predictions into three distinct zygosity classes (Fig. 3f).

More detailed performance data and a complete set of important features can be found in the caption of Fig. 3 and Supplementary Table 2.

To further explore the detection limits of both models in comparison with the TSO500 v2.2.0.2 pipeline, we tested PTEN deletion detection using synthetic data. Both models outperformed the TSO500 pipeline in detecting deletions in in silico data. When tested by synthetically reducing the read depth on PTEN’s 39 bins, our models had a detection limit of 10 bins (Model 1) and 5 bins (Model 2), compared to the TSO500 pipeline’s limit of 20 bins (Fig. 2c and Supplementary Fig. 1).

### Validation and performance comparison

Both models were validated using two independent validation cohorts. Cohort A comprised ten prospective PTEN-deleted samples, identified by TSO500 or visual bin plot analysis after the training/test data freeze. The combined cohort B + C included retrospective samples identified as potentially PTEN deleted by Model 1 from 204 cases of the MASTERsg cohort (excluding the training/test cohort) that were not classified as deleted by the TSO500 pipeline (cohort B, *n* = 33). Cohort C consisted of randomly selected, entity-matched control samples that were uniformly classified as PTEN-WT by the TSO500 pipeline, visual bin plot analysis, and Model 1 (*n* = 31). Because validation cohort B consisted of samples predicted to harbor previously unrecognized PTEN deletions, PTEN IHC was used as a surrogate endpoint reflecting PTEN protein loss.

The median PTEN FC of validation cohort A was 0.53 (range, 0.15–0.59) for the six patient samples identified as PTEN-deleted by the TSO500 pipeline, and 0.88 (range, 0.73–1.05) for the four samples with focal deletions identified by visual bin plot analysis. Bioinformaticians’ zygosity evaluation resulted in homozygous in one of six and heterozygous deletions in five of six samples identified as PTEN-deleted by the pipeline, and homozygous in one of four and heterozygous deletions in three of four samples identified by visual bin plot analysis. PTEN IHC could be performed in eight of the ten samples due to limited tissue availability. Among these cases, the median H-score was 0 (range, 0–10). All four focal deletions demonstrated negative staining, consistent with the expected PTEN-loss phenotype (Supplementary Table 1).

The median PTEN FC of validation cohort B was 0.75 (range, 0.65–0.97) and bioinformaticians’ zygosity evaluation led to a subcategorization of heterozygous deletions in 7 of 33 and WT in 26 of 33 samples. For further analysis, patients 73, 75, and 99 were excluded due to the coexistence of pathogenic PTEN mutations, and patient 78 was excluded due to a failure in quality control of the sequencing data. We performed IHC staining on a subset of 20 samples due to tissue availability. Within this cohort, the median H-score was 10 (range, 0–270). Notably, 12 samples exhibited PTEN protein loss that was concordant with the deletion predictions of Model 1 (H-score range, 0–20). Three samples had inconclusive H-scores ranging from 30 to 40, all within the lower range of the inconclusive category and indicative of markedly reduced PTEN protein expression compared with retained expression (H-score ≥90). Five patient samples showed retained PTEN protein expression, with H-scores ranging from 95 to 270 (Supplementary Table 1).

The median PTEN FC of the WT-control cohort C was 0.99 (range, 0.84–1.26), bioinformaticians’ zygosity evaluation led to a uniform confirmation of WT. The median H-score of the 17 samples available for staining was 80 (range, 0–280). One outlier (patient 108) showed discordant findings, with a PTEN FC of 0.84 and an H-score of 0, suggesting potential post-transcriptional modification. The sample was retained in the cohort (Supplementary Table 1). Patient 91 was included in the WT-control cohort C based on a primary tumor sample that exhibited PTEN WT status. Additionally, a separate liver metastasis biopsy from the same patient, which was obtained prior, was found to have a focal PTEN deletion in Model 1.

More detailed patient data, including full zygosity evaluations and TCC information, are presented in Table 1 and Supplementary Table 1.

Initial analysis of validation cohort B + C revealed significant classification disagreement between visual bin plot analysis and IHC results. Model performance was therefore evaluated against both approaches. Against visual bin plot analysis, Model 1 and Model 2 achieved low F1 scores (0.25 and 0.32, respectively). In contrast, performance substantially improved against IHC (F1 scores of 0.76 and 0.80, respectively; Supplementary Table 2). The training/test cohort used genomic assessment by the TSO500 pipeline, complemented by visual bin plot analysis as the reference to define PTEN deletion status for model training, consistent with the objective of training the models to detect genomic PTEN deletions. IHC provided an independent functional assessment of PTEN protein expression and therefore served as the reference for subsequent validation performance analyses.

To benchmark the CNV-calling performance of Models 1 and 2 against an established read-depth-based CNV caller, both validation cohorts were additionally analyzed using CNVkit^9^.

**Fig. 4:**
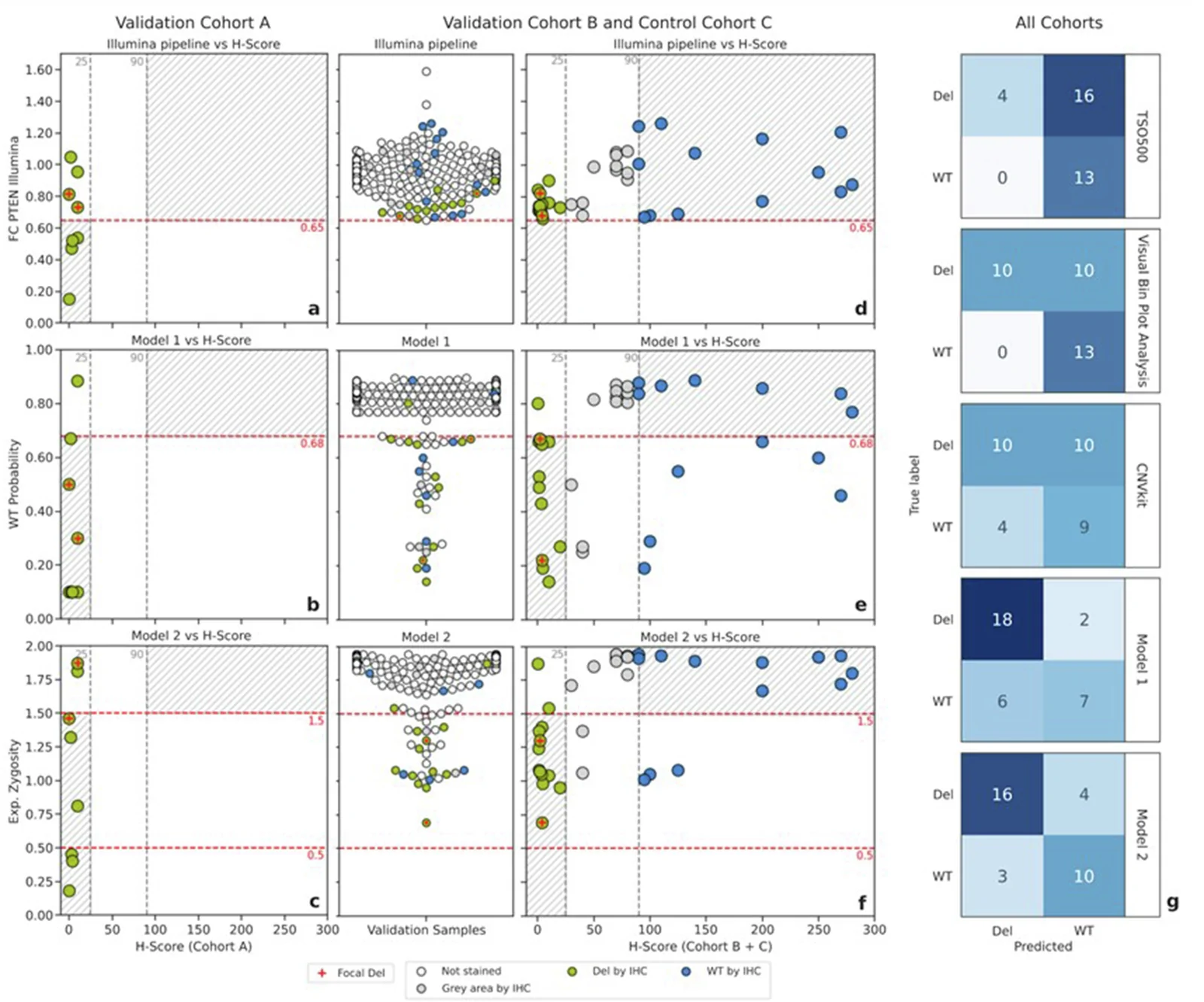
Performance of model prediction on validation cohorts and model interpretability. Colors corresponding to immunohistochemistry (IHC) results (see legend), red cross = focal deletions (Del), dashed red lines = model thresholds, dashed grey lines = H-score thresholds, grey hatching = confirmed deleted and confirmed wild-type (WT) samples, respectively. **a, b, c** Performance on prospective, PTEN deleted validation cohort A; H-score classes vs. prediction. **a** Performance of TSO500 v2.2.0.2 pipeline, achieving a precision of 0.5, correctly identifying 4/8 samples with PTEN protein loss. **b** Performance of Model 1, achieving a precision of 0.88, correctly identifying 7/8 deletion-positive cases. **c** Performance of Model 2, achieving a precision of 0.75, correctly calling 6/8 deletions. Lower dashed red line = threshold for zygosity 0/1 (del), upper red line = threshold for zygosity 1.5 (WT). **d, e, f** Performance on retrospective validation cohort (combined validation cohort B + C). Left panel: MASTERsg cohort including unstained retrospective samples (*n* = 204), right panel: stained samples of validation cohort B + C; H-score classes vs. prediction. **d** Performance of the TSO500 v2.2.0.2. pipeline: false negatives (FN) = 12, true negatives (TN) = 13, true positives (TP) = 0, false positives (FP) = 0, sensitivity = 0, specificity = 1; **e** Performance of Model 1: FP = 6, TN = 7, TP = 11, FN = 1, sensitivity = 0.92, specificity = 0.54. **f** Performance of Model 2: FN = 2, TN = 10, TP = 10, FP = 3, sensitivity = 0.83, specificity = 0.77. **g** Confusion matrices displaying validation cohort classification performance for TSO500, visual bin plot analysis, CNVkit, as well as Model 1 and Model 2. Model 1 achieved the highest sensitivity (0.90), followed by Model 2 (0.80). CNVkit performed comparably to the visual bin plot analysis in terms of sensitivity (0.50), and TSO500 showed the lowest sensitivity (0.20).

In validation cohort A, Model 1 achieved a sensitivity of 0.88 (95% CI, 0.53–0.98) and an F1 score of 0.93, outperforming the TSO500 v2.2.0.2 pipeline with an F1 score of 0.67 (Fig. 4a, b) and CNVkit with an F1 score of 0.77 (Fig. 4g and Supplementary Table 2). For the combined validation cohort B + C using TSO500 v2.2.0.2 data, Model 1 achieved a sensitivity of 0.92 (95% CI, 0.65–0.99), a specificity of 0.54 (95% CI, 0.29–0.77), an F1 score of 0.76, and a Matthew Correlation Coefficient (MCC) of 0.49 (Supplementary Table 2). MCC is a robust and balanced metric that evaluates classification performance by accounting for all four components of the confusion matrix (see Methods section). Compared with Model 1, the TSO500 pipeline and CNVkit showed lower sensitivities of 0.00 (95% CI, 0.00–0.24) and 0.42 (95% CI, 0.19–0.68), respectively, and specificities of 1.00 (95% CI, 0.77–1.00) and 0.69 (95% CI, 0.42–0.87), respectively (Fig. 4d, e, g and Supplementary Table 2). As cohort B was selected based on Model 1 predictions, the comparative performance results for cohort B + C should be considered in the context of this model-based selection.

Model 2 also performed strongly on validation cohort A with a sensitivity of 0.75 (95% CI, 0.41–0.93) and an F1 score of 0.86, demonstrating superior performance compared to the TSO500 pipeline (Fig. 4a, c) and CNVkit (Fig. 4g and Supplementary Table 2). In the combined validation cohort B + C, Model 2 had a sensitivity of 0.83 (95% CI, 0.55–0.95), specificity of 0.77 (95% CI, 0.50–0.92), F1 score of 0.80, and MCC of 0.60, underscoring its enhanced classification quality over the TSO500 pipeline (Fig. 4d, f) and CNVkit, (Fig. 4g and Supplementary Table 2).

To validate the consistency of deletion calling across different TSO500 pipeline versions, sequencing data from the validation cohorts were additionally processed with version 2.6.0.2 of the TSO500 pipeline as model input. The results for validation cohort A remained unchanged among the pipeline versions. In the combined cohort B + C, F1 scores showed a slight decrease, from 0.76 to 0.67 for Model 1 and from 0.80 to 0.72 for Model 2, with sensitivities of 0.67 (95% CI, 0.39–0.86) and 0.75 (95% CI, 0.47–0.91), and specificities of 0.69 (95% CI, 0.42–0.87) and 0.69 (95% CI, 0.42–0.87), respectively (Supplementary Table 2). Importantly, both models reliably detected all focal PTEN deletions in the validation cohort, even with the updated pipeline version (Supplementary Fig. 2).

To further assess the generalizability of our approach, we evaluated TSO500 deletion calling, Model 1, and Model 2 in an independent external validation cohort comprising 61 randomly selected samples from 24 solid tumor entities without preselection based on PTEN status, provided by the Institute of Pathology, School of Medicine and Health, Technical University of Munich, Germany. More detailed patient data, including full zygosity evaluations and TCC information, are shown in Table 1 and Supplementary Table 1.

TSO500 data generated with pipeline version 2.6.0.2 served as input for all analyses. PTEN IHC was performed in 50 cases for which tumor tissue was available; six cases with inconclusive H-scores within the predefined area (>25 to <90) were excluded, leaving 44 cases with conclusive IHC results for performance assessment. Both ML models achieved higher F1 scores than those observed in validation cohort B + C when analyzed using the same TSO500 pipeline version, reaching 0.75 for Model 1 and 0.81 for Model 2 (Supplementary Table 2, Supplementary Fig. 3). Compared with validation cohort B + C, sensitivity remained unchanged for Model 1 at 0.67 (95% CI, 0.44–0.84) and decreased slightly for Model 2 from 0.75 to 0.72 (95% CI, 0.49–0.88), while specificity increased substantially for Model 1 from 0.69 to 0.92 (95% CI, 0.76–0.98) and for Model 2 from 0.69 to 0.96 (95% CI, 0.81–0.99) (Supplementary Table 2 and Supplementary Fig. 3). In comparison, TSO500 deletion calling achieved an F1 score of 0.71, with a sensitivity of 0.56 (95% CI, 0.34–0.75) and a specificity of 1.00 (95% CI, 0.87–1.00) (Supplementary Table 2 and Supplementary Fig. 3).

To further investigate the possible biological relevance of the decision criteria used by the models, we performed SHapley Additive exPlanations (SHAP) value analysis to identify the contribution of every feature to individual model predictions (Supplementary Fig. 4a, b; see “Methods” section). Model 1 focused on raw counts from specific bins, as indicated by SHAP values greater than zero, while derived features, for example, representing the slope of the bin count graph at a certain position, did not contribute meaningfully to the predictions. For Model 2, however, derived features, such as log-transformed polynomial values and the derivatives of the bin count graph, contributed to the prediction (Supplementary Fig. 4a). For consistency, comparisons of relevant bins between models were restricted to the raw bin counts. The SHAP analysis revealed the overlap of several high-impact bins for both models with known exonic or enhancer regions, as defined by the *Ensembl project*^10^. For Model 1, six specific features (bins 139, 141, 153, 158, 159, and 162) drove predictions. Most were intronic, but bin 159 was exonic, and bin 139 aligned with a known enhancer region (Supplementary Fig. 4b). For Model 2, using the same feature selection criteria, 12 bins were identified as the most impactful (bins 136, 138, 139, 141, 144, 146, 147, 159, 160, 161, 170, and 174). Among these, bins 136, 159, and 174 corresponded to exonic regions and bin 139 with an enhancer region (Supplementary Fig. 4b).

More detailed performance data and a complete set of important features can be found in the caption of Fig. 4, Supplementary Table 2, and Supplementary Figs 2, 3, and 4.

## Discussion

Molecularly informed therapy is gaining increasing importance in the treatment of advanced tumors. Since panel sequencing is the most common method of molecular diagnostics, improving the sensitivity to reliably detect addressable targets is highly relevant. Copy number variations, such as amplification of ERBB or MET receptors and deletions of CDKN2A/B and PTEN, are regularly utilized biomarkers for targeted therapy approaches^11^. While Conroy et al. found a positive predictive value of 100% for nonfocal PTEN loss with FC below 0.7^12^, Giacò et al. confirmed the risk of missing focal deletions^4^, reinforcing the need to extend TSO500 CNV calling. By visualizing the TSO500 CNV caller’s raw data for over 500 genes for focal deletion identification, we were able to detect a relevant number of focal deletions missed by the standard pipeline. To automate this visual bin plot analysis, we developed ML models for both focal deletion detection (Model 1) and zygosity evaluation (Model 2), using the PTEN gene as an example. Both models showed high sensitivity and selectivity in discriminating between deleted and wild-type PTEN cases. Model 1 successfully categorized all but one test patient (12/13), including every focal deletion missed by the TSO500 pipeline, markedly improving PTEN deletion detection.

Most notably, Model 1 uncovered 33 additional potential PTEN deletions in patient samples previously considered unremarkable by the TSO500 pipeline (validation cohort B), 18 of which were also not reported by the established read-depth CNV caller CNVkit. This finding was supported by absent or markedly reduced PTEN protein expression in 75% (15/20) of cases with tumor tissue available for staining, supporting the ability of Model 1 to identify additional candidate PTEN deletions not detected by the TSO500 pipeline or CNVkit that may have therapeutic relevance. These findings suggest that machine-learning approaches may enhance deletion calling in panel sequencing and raise the question of which unaccounted genomic features are critical determinants of PTEN expression.

However, because validation cohort B was intentionally enriched for samples flagged by Model 1, it was not designed to estimate prevalence-adjusted performance metrics such as positive predictive value or false-positive burden in an unselected clinical population. Moreover, selection based on Model 1 predictions inherently favors Model 1 in comparative performance analyses within this cohort, precluding an unbiased head-to-head comparison with Model 2 and conventional CNV calling approaches. To address this limitation, we evaluated both Model 1 and Model 2 in an independent validation cohort from an external source sequenced with TSO500 and processed with the pipeline version 2.6.0.2. The cohort consisted of randomly selected patients with a variety of solid tumor entities and had not been preselected based on PTEN status. Both models maintained favorable overall performance and achieved higher sensitivity than the TSO500 pipeline. Compared with validation cohort B + C analyzed using the same TSO500 pipeline version (v2.6.0.2), specificity increased substantially in the external validation cohort (Model 1: 0.69 vs. 0.92; Model 2: 0.69 vs. 0.96), while sensitivity remained largely unchanged for both models (Model 1: 0.67 vs. 0.67; Model 2: 0.75 vs. 0.72). These findings suggest that the models capture biologically relevant signals that generalize beyond the training institution and are not solely dependent on cohort-specific characteristics. Overall, these findings support the technical robustness and transferability of the approach across institutions and patient populations. Nevertheless, larger prospective studies in consecutive patient cohorts will be required to determine real-world performance characteristics and clinical utility beyond this proof-of-concept study.

While Model 2 demonstrated good overall performance for the test set, the lower scores for zygosity 2 suggest that accurate zygosity classification remains more challenging than binary classification. This is likely due to the small dataset and extra complexity in distinguishing between the three zygosity states compared to binary deletion detection. Model 2 predictions showed good concordance with PTEN IHC status, separating most samples with PTEN protein loss from those retaining PTEN expression. Model 2 predictions in relation to IHC staining separated the samples into true positives and true negatives. Detection of deletions in the validation cohort was comparable to that of Model 1, with Model 2 identifying one fewer deletion while yielding fewer false positives. In a clinical setting, the use of Model 2 for deletion calls, followed by IHC validation of only positive cases, would reduce validation efforts despite slightly reduced sensitivity.

Taken together, since both models showed a modest increase in false-positive calls compared with standard TSO500 deletion calling, they should primarily be considered screening tools rather than stand-alone methods for definitive PTEN status assignment. Further methodological refinement and prospective validation will be required before sequencing-based PTEN deletion calling alone can be considered sufficiently conclusive for routine clinical use.

To gain insight into the features driving model predictions, we performed SHAP analysis. Several high-impact bins overlapped with known exonic or enhancer regions. While this observation may suggest potential biological relevance, the overlap was limited and should be interpreted cautiously, given the relatively small dataset. Larger studies will be required to determine whether these regions are consistently enriched among influential features. Notably, several impactful SHAP features aligned with known PTEN enhancer regions, whereas many other key bins localized to intronic areas, which could indicate additional regulatory elements but warrant further investigation.

The limited size of the training cohort and extensive hyperparameter optimization raise the possibility of overfitting. This risk was mitigated through strong regularization, including row and column subsampling, L1/L2 penalties, and early stopping, resulting in a sparse Model 1 ensemble utilizing only 6 of 119 available features. Performance was assessed using cross-validation, a held-out test set, and independent validation cohorts. Despite the limited sample size, the consistent performance across unseen cohorts provides preliminary support for model generalizability. Larger cohorts will be required for more precise performance estimates and threshold calibration. However, retraining might be useful when pipeline changes accumulate over time. We compared two TSO500 pipeline versions, v2.2.0.2 and v2.6.0.2, and observed a moderate drop in model performance (Model 1: F1 score 0.76 vs. 0.67; Model 2: 0.80 vs. 0.72). While the models consistently displayed enhanced deletion detection compared to both TSO500 pipeline versions, minor classification differences emerged when models were run on data from the pipeline on which they had not been trained.

While our models enhance the genomic detection of PTEN deletions, this does not necessarily reflect PTEN protein loss, because copy number variations are only one of several drivers of PTEN inactivation, which is also modulated by point mutations, promoter hypermethylation, or microRNA-mediated silencing, alongside complex post-transcriptional and post-translational regulatory mechanisms^13^. This is a known limitation of DNA sequencing and can only be addressed by including information derived from transcriptome^11^ and proteome analytics^14^. However, multi-omics approaches are currently restricted to a few precision oncology programs for a limited number of cancer patients.

A notable limitation of our study is the inability to completely exclude subclonal PTEN deletions, which may influence signal interpretation. Panel sequencing data derived from bulk tumor tissue reflects an average signal, where subclonal events can produce attenuated fold-change values, complicating the distinction between true focal deletions and technical noise^15^. While our models incorporated tumor cell content provided by expert pathological review as an input parameter to account for sample purity, and Model 2 was designed to differentiate between heterozygous and homozygous states, these approaches remain inherently limited by the nature of bulk sequencing. Future iterations would benefit from incorporating computational clonality assessments^16^ to further refine the interpretation of genomic signal strength and its relationship to the heterogeneity of protein loss observed in IHC. In this context, the observed concordance between model-predicted deletions and PTEN protein loss suggests that genomic features captured by the models may reflect biologically relevant PTEN disruption, representing a meaningful step toward bridging genomic and functional readouts in routine diagnostics. The same considerations also apply to the interpretation of our in silico deletion analysis, which cannot account for the biological and technical factors underlying attenuated copy-number signals in clinical samples. Artificial reduction of read depth across defined PTEN bins represents a simplified model that does not fully recapitulate the conditions encountered in clinical samples, including the potential subclonal occurrence of focal deletions^17^, variable tumor purity, GC-related coverage bias^18^, sequencing noise, and differences in sample quality. Accordingly, the observed in silico detection limits should be interpreted as comparative technical estimates rather than absolute limits of detection in clinical samples. IHC was used as an orthogonal validation method to assess PTEN protein expression, rather than as a direct measure of genomic deletion. The training/test reference labels were derived from genomic assessment using the TSO500 pipeline complemented by visual bin plot analysis. During validation, model performance varied depending on the evaluation reference, with low agreement with visual bin plot analysis and notably higher concordance with IHC. As an independent and clinically established functional readout of PTEN protein expression, H-score was therefore used as the reference for performance evaluation in the validation cohorts. The lower concordance of visual bin plot analysis with IHC observed during validation highlights a limitation of the sequencing-derived training labels and underscores the value of orthogonal validation. However, IHC itself has limitations, including its single-gene implementation, restricted antibody availability hindering large-scale application, and its indirect reflection of genomic alterations, which became evident in the staining of the WT-cohort C, with one patient showing negative PTEN staining and 7 of 16 patients showing an inconclusive staining result. Although showing its potential in this study, IHC has limitations when planning to extend it to further genes or the complete scope of the panel. Other publications validating CNV-calling enhancement tools have employed experimental approaches, including quantitative PCR, multiple ligation-dependent probe amplification, and array comparative genomic hybridization^19^, as well as computational validation strategies based on curated reference datasets^5^, and in silico simulated CNVs^20^. These complementary approaches represent alternatives that we intend to explore in future work.

In conclusion, our ML models significantly improved the sensitivity of PTEN deletion detection, uncovering additional therapeutically targetable alterations in cancer patients with limited or no further therapeutic options. This highlights a crucial clinical significance, as demonstrated by the N^2^M^2^ umbrella trial for glioblastoma, in which the temsirolimus arm for patients with activated mTOR signaling was the only therapy arm to benefit from matched targeted therapy^21^. Although no secondary FC threshold was defined to reduce reliance on IHC, the potential to identify additional clinically relevant deletions may justify the added diagnostic effort and support the further evaluation and integration of ML-assisted CNV analysis. Larger training and validation cohorts are required for further model optimization. Efforts are underway to extend this method to detect deletions in other genes.

## Methods

This study details the development and validation of two novel ML models designed to improve the detection of PTEN deletions from TSO500 panel data. The design utilized both retrospective and prospective patient cohorts enrolled in the MASTERsg precision oncology program at the NCT/UCC in Dresden, Germany, as well as an external validation cohort from the Institute of Pathology, School of Medicine and Health, Technical University of Munich, Germany. Biological validation was conducted via IHC.

### Ethical approval

This study was conducted in accordance with the principles of the 1964 Declaration of Helsinki. All 132 participating patients of the MASTERsg cohort provided written informed consent for the banking of tumor and control tissues, molecular analysis, and collection of clinical data under a protocol approved by the Ethics Committee of the Technical University Dresden, ethics approval number EK431102015. The external validation cohort comprised 61 patients from the Institute of Pathology, School of Medicine and Health, Technical University of Munich, Germany. All patients in this cohort provided written informed consent for the banking of tumor and control tissues, molecular analysis, and collection of clinical data under protocols approved by the local Ethics Committee of the Technical University of Munich.

### Study cohorts and data collection

All 133 study patients of the training/test cohort and validation cohorts A, B, and C had advanced cancers, including multiple solid tumor entities and a single hematologic malignancy (see Supplementary Table 1). All were sequenced using the TSO500 DNA assay within the MASTERsg program. The MASTERsg program enrolls adult patients aged 18 years or older of all genders with advanced or metastatic solid or hematologic cancer for whom available approved systemic therapy options are limited or have been exhausted, and who require identification of further systemic treatment options. Informed consent was obtained from all patients for tumor and normal tissue sequencing, as well as for further processing and reuse of sequencing and clinical data for research purposes. For ML model development, patients were subcategorized into a training/test cohort and a validation cohort, which were further divided into subcohorts A, B, and C. Median PTEN FCs and TCCs were evaluated for each (sub)cohort. Additionally, a zygosity evaluation (0 = homozygous deletion, i.e., both alleles deleted; 1 = heterozygous deletion, i.e., one allele deleted; 2 = WT) was conducted by the program’s bioinformaticians. After panel sequencing, tumor samples from all study patients were IHC-stained for PTEN.

The training/test cohort consisted of 59 patients with progressed solid tumors underwent sequencing using the TSO500 DNA assay and were subcategorized based on PTEN status. Twenty-nine patients exhibited PTEN deletions, as identified by the TSO500 pipeline using an FC cutoff of <0.65. These patients represented 11 distinct tumor subgroups. Five patients had focal PTEN deletions that were not detected by the pipeline due to FC values exceeding the 0.65 threshold, but were identified by the program’s bioinformaticians through visual bin plot analysis. These patients originated from four tumor entities. The remaining 25 patients were classified as PTEN WT and were distributed across 17 tumor entities. For further patient details, see Supplementary Table 1.

Model validation was conducted on two subgroups of patients with progressed hematologic and solid cancers sequenced using the TSO500 DNA assay: Validation cohort A (*n* = 10) included six cases with PTEN deletions identified by the TSO500 pipeline and four cases identified as deleted via visual bin plot analysis after the data freeze for the training/test cohort. These patients represented eight distinct tumor subgroups. The combined validation cohort B + C consisted of validation cohort B (*n* = 33), which included cases from a retrospective re-analysis of 204 MASTERsg samples not identified as PTEN-deleted by the TSO500 pipeline but flagged as potentially deleted by Model 1. The patients in this cohort originated from 14 groups of tumor entities. Cohort C (*n* = 31) consisted of randomly selected, entity-matched patient samples from the same re-analysis, all uniformly classified as PTEN-WT by the TSO500 pipeline, visual bin plot analysis, and Model 1. These patients represented 11 tumor entity subgroups. For further patient details, see Supplementary Table 1.

To evaluate the ML models’ generalizability, additional validation was conducted on an external cohort consisting of 61 patients with advanced cancers of 24 solid tumor entities, sequenced with the TSO500 DNA assay by the Institute of Pathology, School of Medicine and Health, Technical University of Munich, Germany. Patients were randomly selected without preselection based on PTEN status. Visual bin plot analysis and zygosity evaluation were conducted by the MASTERsg program’s bioinformaticians in Dresden. Corresponding tumor tissue samples, which were available for 50 patients, were IHC-stained for PTEN by the MASTERsg program’s pathologists in Dresden. Bioinformaticians were blinded to IHC results, and pathologists were blinded to TSO500 sequencing data and visual bin plot analysis. For further patient details, see Supplementary Table 1.

### Panel sequencing

For targeted sequencing, 40–120 ng of the total DNA was used. Library preparation was performed using TruSight Oncology 500 Panel (Illumina, San Diego, CA, USA) according to the manufacturer’s protocol^3^. Barcoded libraries were sequenced (2 × 150 bp paired-end) on an Illumina NextSeq 500 or 550 platform. Raw sequencing data were analyzed using either the Illumina TSO500 Local App version 2.2.0.2 in a Docker container or the current DRAGEN TSO500 version 2.6.0.2 pipeline, with GRCh37 serving as the reference genome. In addition to the CNV results provided by Illumina, enhanced CNV analysis was performed by analyzing the raw data from Illumina’s CNV Caller, hereinafter referred to as ‘visual bin plot analysis’. Normalized bin counts for each gene, dividing the gene into an individual number of bins comprising sequencing regions between 50 and 250 bp, as provided by the TSO500 pipeline, were plotted against an internal in-house reference line, corresponding to the median normalized count of each bin derived from 39 reference samples sequenced previously. The PTEN locus was classified as WT or (focally) deleted, according to the distribution of the normalized bin count in contrast to the internal in-house reference, taking estimated TCC into account. Bioinformaticians were blinded to the IHC classification.

To demonstrate the algorithmic improvement at the exon-level, the DRAGEN TSO500 mapping results of the internal validation cohorts were processed using CNVkit, an established read-depth CNV caller designed for use with hybrid capture^9^. CNVkit 0.9.12 was executed with the default parameters. A total of fifteen normal samples were provided to establish a baseline reference. Regions that exhibited significant variability, as classified by UCSC (provided by CNVkit), were excluded. Notably, none of these highly variable regions coincided with the PTEN region. Called CNVs were classified as “deletion”, “focal deletion” or “WT” based on whether all bins, some bins or no bins with copy numbers below 2 were reported, respectively.

### Generation of in silico data

To simulate various PTEN deletion scenarios, a BAM file from a patient with WT PTEN was used as the starting point. Based on the PTEN target regions defined in the TSO500 panel manifest (BED file), in silico deletion patterns were generated by selectively removing 50% or 100% reads from 2 to 30 bins in contiguous stretches. BAM file manipulation was performed using SAMtools (v1.17) for read filtering, sorting, indexing, FASTQ conversion, and BEDtools (v2.31.0) for genomic region-based operations. The resulting BAM files were sorted and indexed, and paired-end FASTQ files were extracted using SAMtools fastq to enable downstream sequencing analysis of the manipulated datasets.

### Immunohistochemical staining of PTEN

After genetic analysis, IHC staining for PTEN (QR042; Quartett, Potsdam, GER; monoclonal, dilution 1:200) was prepared from 4 µm sections of formalin-fixed paraffin-embedded tumor samples. The samples were analyzed microscopically, and the staining intensity and percentage of positively stained cells were quantified by two board-certified pathologists who independently examined the staining. From these data, the H-score, a quantitative assessment of IHC, was calculated using the following formula: percentage of weak nuclear and/or cytoplasmic staining + percentage of moderate staining × 2 + percentage of strong staining × 3, as previously described^22,23^. Conclusively, score values range from 0 (completely negative) to 300 in cases with complete and strong expression of PTEN protein in neoplastic cells. Owing to the biological heterogeneity of the analyzed tumor entities, the application of rigid cutoffs for PTEN expression can be challenging. In practice, IHC evaluation is guided by comparison with adjacent normal tissue and on-slide controls. Nonetheless, for this study, we applied practical thresholds: tumors with weak staining in most tumor cells or strong staining in ≥30% of cells (H-score ≥90) were considered positive, whereas tumors with only weak focal staining (up to 25% of cells) or isolated strong signals (H-score ≤25) were classified as negative. Intermediate values (>25 to <90) were considered inconclusive. Pathologists were blinded to the TSO500 sequencing data.

### Feature engineering

To optimize the capture of PTEN copy number alterations, we engineered a set of features from normalized bin counts, including the FC output from the TSO500 pipeline and TCC, given that low tumor purity can attenuate the deletion signal. To enable the model to capture non-linear patterns indicative of PTEN deletions, we generated polynomial features from the normalized bin counts. Specifically, we computed a log-transformed polynomial fit across the PTEN region and included its first derivative to capture the local slope at each bin position. This approach was based on the hypothesis that clusters of consecutively low bins are more indicative of a true deletion than isolated low values. In total, each tumor sample was represented by a feature vector comprising normalized bin count reads, log-transformed polynomial and derivative values, as well as global FC and TCC. This comprehensive feature set provides the classifier with sufficient discriminatory power to distinguish true deletions from background noise.

### Model development

We developed two Extreme Gradient Boosting (XGBoost) classifiers^8^: Model 1 for binary classification (PTEN deletion vs WT) and Model 2 for three-class prediction of PTEN zygosity (homozygous deletion, heterozygous deletion, or WT). XGBoost is a three-based classification ensemble method known for its efficiency and high accuracy on structured data. The binary Model 1 outputs the probability of a WT PTEN status, and the multiclass Model 2 outputs a probability distribution across the three zygosity states. The motivation to separate the binary model was to maximize sensitivity for any deletion event, whereas the multiclass model was designed to further discriminate between partial or complete deletions, providing additional biological insight. We used Python’s XGBoost library with a logistic objective for the binary model and a softmax objective for the multiclass model. To address class imbalance, we applied higher weights to deletion-positive samples in the binary loss function. No class weighting was applied to the multiclass model. Given the limited training dataset size of 43 labeled cases, we employed a stratified cross-validation (CV) strategy, ensuring each fold included one deletion-positive sample to maximize data usage and reduce overfitting. Performance metrics were averaged across all folds and used for hyperparameter tuning. Each fold was used once for validation while the model was trained on the remaining data, resulting in *n* cross-validation trials.

This approach, similar to leave-one-out CV, ensured each deletion-positive sample was represented and provided a robust performance estimate. The averaged metrics across folds were used to guide hyperparameter optimization. We applied Bayesian optimization via the *Syne Tune* library (AWS Labs) to efficiently explore hyperparameters such as learning rate, max tree depth, and regularization to maximize AP (binary model) and mean AP (multiclass model). Around 1000 configurations were evaluated per model, and the best-performing setup was used for final training on the full dataset. To limit overfitting given the small cohort size and extensive hyperparameter optimization, we applied row and column subsampling (70%), L1/L2 regularization, minimum child-weight constraints, and early stopping. Model 1 converged to a sparse ensemble of 16 trees using only 6 of 119 available features, whereas Model 2 showed greater complexity, likely reflecting the more challenging three-class classification task. Performance was evaluated using cross-validation, a held-out test set, and an independent external validation cohort.

### Threshold selection

For Model 1, the deletion threshold was selected on the training set as the probability cutoff that maximizes the F1 score. This threshold was subsequently applied unchanged to the independent test set and external validation cohorts to assess generalizability. The F1 score is the harmonic mean of precision and recall, providing a balance between these two metrics, especially useful when dealing with imbalanced classes. The F1 score is defined as:

1 $${{F}}1=\frac{2\times {Precision}\times {Recall}}{{Precision}+{Precision}}$$

For Model 2 we transformed the softmax output into a single, continuous expected zygosity score by summing the class probabilities for each class weighted by their respective zygosity values (0, 1, and 2):

2 $${Expected}\,{Zygosity}=0\times \rho \left(0\right)+1\times \rho \left(1\right)+2\times \rho$$

We assigned weights of 0, 1, and 2 to maintain the natural ordering of gene copy states. We then converted the expected value back to a discrete class using two default thresholds. These thresholds (0.5 and 1.5) split the continuous scale into three segments corresponding to the original categorical labels. For analytical purposes, values below 1.5 were considered as being deleted.

### Evaluation metrics

We evaluated the binary classifier on the independent test set of 16 samples. Average precision (AP), equivalent to PR AUC, served as the primary metric for model selection during hyperparameter selection, as it is particularly sensitive to the positive (del) class and informative under class imbalance. For test and validation evaluation, we also report the ROC AUC, which provides a threshold-independent measure of overall discrimination between deletion and WT. F1 was used at the chosen decision threshold to assess binary performance^24^.

Additionally, the Matthew Correlation Coefficient (MCC) was computed^25^. MCC is the robust metric that considers all four values in the confusion matrix (true positive, true negative, false positive and false negative), producing a high score only if the prediction obtained good results in all four categories. Furthermore, it is particularly useful for imbalanced datasets. MCC is defined as:

3 $${MCC}=\frac{{TP}\times {TN}-{FP}\times {FN}}{\sqrt{({TP}+{FP})({TP}+{FN})({TN}+{FP})({TN}+{FN})}}$$

To quantify uncertainty, 95% confidence intervals (CI) were calculated using the Wilson score interval method where applicable.

### Interpretability analysis

A key advantage of using gradient-based models is their inherent interpretability. To understand the model decision-making, we utilized “TreeExplainer” from SHapley Additive exPlanations (SHAP) analysis^26^. SHAP assigns an additive contribution to every feature for each individual prediction, allowing for the examination of both local (sample-wise) and global (dataset-level) effects. For every validation sample, we computed SHAP values and then aggregated them (mean |SHAP|) to obtain a ranked list of feature influences. Features whose SHAP values were zero were discarded, as they do not contribute to the decision process. To ensure a fair comparison between the models, we restricted the analysis to the raw bin counts, the only influential feature type shared by both models.

## Supplementary information

Supplementary_Information

Supp_Table_1

## Data Availability

The sequencing data generated are deposited and available through the European Genome-Phenome Archive (EGA) under the study identifier: EGAS00001008324 - Machine Learning Uncovers Previously Undetectable Genetic Deletions in Cancer Panel Sequencing. ML models and documentation are available via https://github.com/Valavanca/NGS-gene-loss-detection.
