# Supplementary material for: Machine learning uncovers previously undetectable genetic deletions in cancer panel sequencing
Source: NPJ Precis Oncol. 2026 Oct 16;10:397. doi: 10.1038/s41698-026-01756-9 (PMC13675215; doi:10.1038/s41698-026-01756-9)
Supplement: Supplementary file 1 [file 41698_2026_1756_MOESM1_ESM.pdf]

Supplementary Information

In order of appearance in the manuscript:

Supplementary Table 1 (excel file uploaded separately) Patient-level clinicopathological and molecular characteristics of the training/test cohort, validation cohorts A–C, and the external validation cohort, including PTEN fold-change (FC), PTEN deletion status determined by the TSO500 pipeline, visual bin plot analysis, Model 1, Model 2, CNVkit (where applicable), PTEN immunohistochemistry (H-score), and associated clinical annotations

Supplementary Figure 1 TSO500 v2.2.0.2 PTEN normalized bin plot analyses. Set of generated in silico samples with increasing amounts of deleted bins (100% deletion), called by either the TSO500 pipeline, Model 1 (M1) and Model 2 (M2) as PTEN deleted. # del bins: number of deleted bins; for plot description see Fig. 1a.

**# del bins: 0**	**# del bins: 5**	**# del bins: 10**	**# del bins: 15**	**# del bins: 20**	**# del bins: 25**	**# del bins: 30**
FC 0.978	FC 0.950	FC 0.947	FC 0.903	FC 0.152	FC 0.154	FC 0.158
TSO500 WT	TSO500 WT	TSO500 WT	TSO500 WT	TSO500 **DEL**	TSO500 **DEL**	TSO500 **DEL**
M1 WT	M1 WT	M1 **DEL**	M1 **DEL**	M1 **DEL**	M1 **DEL**	M1 **DEL**
M2 WT	M2 **DEL**	M2 **DEL**	M2 **DEL**	M2 **DEL**	M2 **DEL**	M2 **DEL**

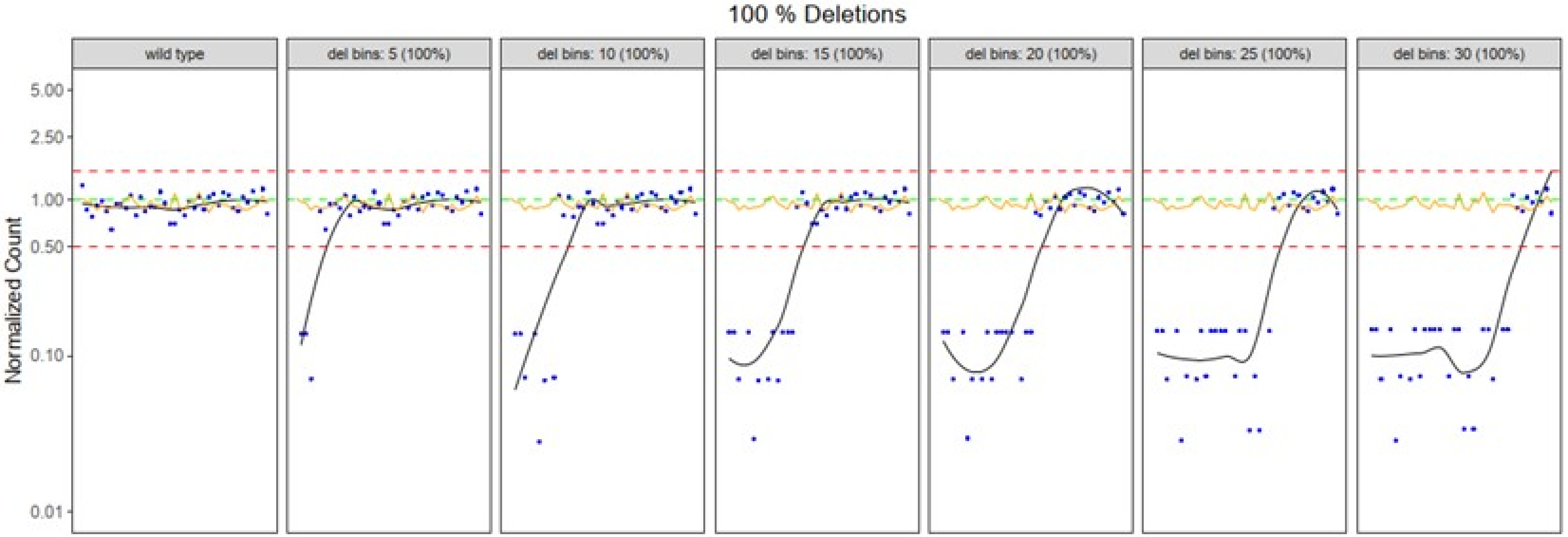

Supplementary Table 2 Performance metrics of the TSO500 pipeline, CNVkit, Model 1 (M1), and Model 2 (M2) across the test set, internal validation cohorts, and the external validation cohort. For the training/test cohort, performance was evaluated against the bioinformaticians' visual bin plot analysis. For validation cohorts, performance was assessed using PTEN immunohistochemistry (H-score) as the reference standard where available. Metrics include sensitivity, specificity, precision, false-positive rate, false-negative rate, negative predictive value, false discovery rate, accuracy, prevalence, F1 score, balanced accuracy, Matthews correlation coefficient (MCC), and average precision (AP). AP and multiclass AP are reported only for the training/test cohort, where probability outputs were available. 95% confidence intervals (CIs) for sensitivity, specificity, and precision were calculated using the Wilson score interval. NaN, metric not applicable because only one outcome class was present in the reference dataset.

	A	B	C	D	E	F	G	H	I	J	K	L
1		TSO500 v2.2.0.2 (reference: visual bin plot analysis)					TSO500 v2.2.0.2 (reference: H-Score)					
2		Test set					Cohort A			Cohort B+C		
3	**Metric**	**TSO500**	**M1**	**M2 Zyg. 0**	**M2 Zyg. 1**	**M2 Zyg. 2**	**TSO500**	**M1**	**M2**	**TSO500**	**M1**	**M2**
4	FN	1	0	1	2	0	4	1	2	12	1	2
5	TN	3	3	10	7	12	0	0	0	13	7	10
6	TP	6	12	4	6	3	4	7	6	0	11	10
7	FP	6	1	1	1	1	0	0	0	0	6	3
8	Sensitivity	0.86	1.00	0.80	0.75	1.00	0.50	0.88	0.75	0.00	0.92	0.83
9	Sensitivity, 95%CI	[0,49, 0,97]	[0,76, 1,00]	[0,38, 0,96]	[0,41, 0,93]	[0,44, 1,00]	[0,22, 0,78]	[0,53, 0,98]	[0,41, 0,93]	[0,00, 0,24]	[0,65, 0,99]	[0,55, 0,95]
10	Specificity	0.33	0.75	0.91	0.88	0.92	NaN	NaN	NaN	1.00	0.54	0.77
11	Specificity, 95%CI	[0,12, 0,65]	[0,30, 0,95]	[0,62, 0,98]	[0,53, 0,98]	[0,67, 0,99]	[NaN, NaN]	[NaN, NaN]	[NaN, NaN]	[0,77, 1,00]	[0,29, 0,77]	[0,50, 0,92]
12	Precision	0.50	0.92	0.80	0.86	0.75	1.00	1.00	1.00	NaN	0.65	0.77
13	Precision, 95%CI	[0,25, 0,75]	[0,67, 0,99]	[0,38, 0,96]	[0,49, 0,97]	[0,30, 0,95]	[0,51, 1,00]	[0,65, 1,00]	[0,61, 1,00]	[NaN, NaN]	[0,41, 0,83]	[0,50, 0,92]
14	False positive rate	0.67	0.25	0.09	0.13	0.08	NaN	NaN	NaN	0.00	0.46	0.23
15	False negative rate	0.14	0.00	0.20	0.25	0.00	0.50	0.13	0.25	1.00	0.08	0.17
16	Negative predictive value	0.75	1.00	0.91	0.78	1.00	0.00	0.00	0.00	0.52	0.88	0.83
17	False discovery rate	0.50	0.08	0.20	0.14	0.25	0.00	0.00	0.00	NaN	0.35	0.23
18	Accuracy	0.56	0.94	0.88	0.81	0.94	0.50	0.88	0.75	0.52	0.72	0.80
19	Prevalence	0.44	0.75	0.31	0.50	0.19	1.00	1.00	1.00	0.48	0.48	0.48
20	F1 score	0.63	0.96	0.80	0.80	0.86	0.67	0.93	0.86	NaN	0.76	0.80
21	Balanced accuracy	0.60	0.88	0.85	0.81	0.96	NaN	NaN	NaN	0.50	0.73	0.80
22	Matthews correlation coefficient	0.22	0.83	0.71	0.63	0.83	NaN	NaN	NaN	NaN	0.49	0.60
23	Average Precision (AP)	NaN	0.96	0.93	0.90	0.64	NaN	NaN	NaN	NaN	NaN	NaN
24	Multiclass AP	NaN	NaN		0.81		NaN	NaN	NaN	NaN	NaN	NaN
25												
26												
27												
28	M1 = Model 1											
29	M2 = Model 2											

	A	M	N	O	P	Q	R	S	T	U	V	W
1		TSO500 v2.6.0.2 (reference: H-Score)										
2		Cohort A				Cohort B+C				ext. Validation		
3	**Metric**	**TSO500**	**CNVkit**	**M1**	**M2**	**TSO500**	**CNVkit**	**M1**	**M2**	**TSO500**	**M1**	**M2**
4	FN	4	3	1	2	12	7	4	3	8	6	5
5	TN	0	0	0	0	13	9	9	9	26	24	25
6	TP	4	5	7	6	0	5	8	9	10	12	13
7	FP	0	0	0	0	0	4	4	4	0	2	1
8	Sensitivity	0.50	0.63	0.88	0.75	0.00	0.42	0.67	0.75	0.56	0.67	0.72
9	Sensitivity, 95%CI	[0,22, 0,78]	[0,31, 0,86]	[0,53, 0,98]	[0,41, 0,93]	[0,00, 0,24]	[0,19, 0,68]	[0,39, 0,86]	[0,47, 0,91]	[0,34, 0,75]	[0,44, 0,84]	[0,49, 0,88]
10	Specificity	NaN	NaN	NaN	NaN	1.00	0.69	0.69	0.69	1.00	0.92	0.96
11	Specificity, 95%CI	[NaN, NaN]	[NaN, NaN]	[NaN, NaN]	[NaN, NaN]	[0,77, 1,00]	[0,42, 0,87]	[0,42, 0,87]	[0,42, 0,87]	[0,87, 1,00]	[0,76, 0,98]	[0,81, 0,99]
12	Precision	1.00	1.00	1.00	1.00	NaN	0.56	0.67	0.69	1.00	0.86	0.93
13	Precision, 95%CI	[0,51, 1,00]	[0,57, 1,00]	[0,65, 1,00]	[0,61, 1,00]	[NaN, NaN]	[0,27, 0,81]	[0,39, 0,86]	[0,42, 0,87]	[0,72, 1,00]	[0,60, 0,96]	[0,69, 0,99]
14	False positive rate	NaN	NaN	NaN	NaN	0.00	0.31	0.31	0.31	0.00	0.08	0.04
15	False negative rate	0.50	0.38	0.13	0.25	1.00	0.58	0.33	0.25	0.44	0.33	0.28
16	Negative predictive value	0.00	0.00	0.00	0.00	0.52	0.56	0.69	0.75	0.76	0.80	0.83
17	False discovery rate	0.00	0.00	0.00	0.00	NaN	0.44	0.33	0.31	0.00	0.14	0.07
18	Accuracy	0.50	0.63	0.88	0.75	0.52	0.56	0.68	0.72	0.82	0.82	0.86
19	Prevalence	1.00	1.00	1.00	1.00	0.48	0.48	0.48	0.48	0.41	0.41	0.41
20	F1 score	0.67	0.77	0.93	0.86	NaN	0.48	0.67	0.72	0.71	0.75	0.81
21	Balanced accuracy	NaN	NaN	NaN	NaN	0.50	0.55	0.68	0.72	0.78	0.79	0.84
22	Matthews correlation coefficient	NaN	NaN	NaN	NaN	NaN	0.11	0.36	0.44	0.65	0.62	0.72
23	Average Precision (AP)	NaN	NaN	NaN	NaN	NaN	NaN	NaN	NaN	NaN	NaN	NaN
24	Multiclass AP	NaN	NaN	NaN	NaN	NaN	NaN	NaN	NaN	NaN	NaN	NaN
25												
26												
27												
28	M1 = Model 1											
29	M2 = Model 2											

Supplementary Figure 2 Cross-version stability of model predictions from the TSO500 pipeline. Scatterplots comparison of all validation samples from the TSO500 v2.2.0.2 (x-axis, training version) to v2.6.0.2 (y-axis) for Model 1 (**a**) and Model 2 (**b**). Red dashed lines are appropriate model-specific decision thresholds.

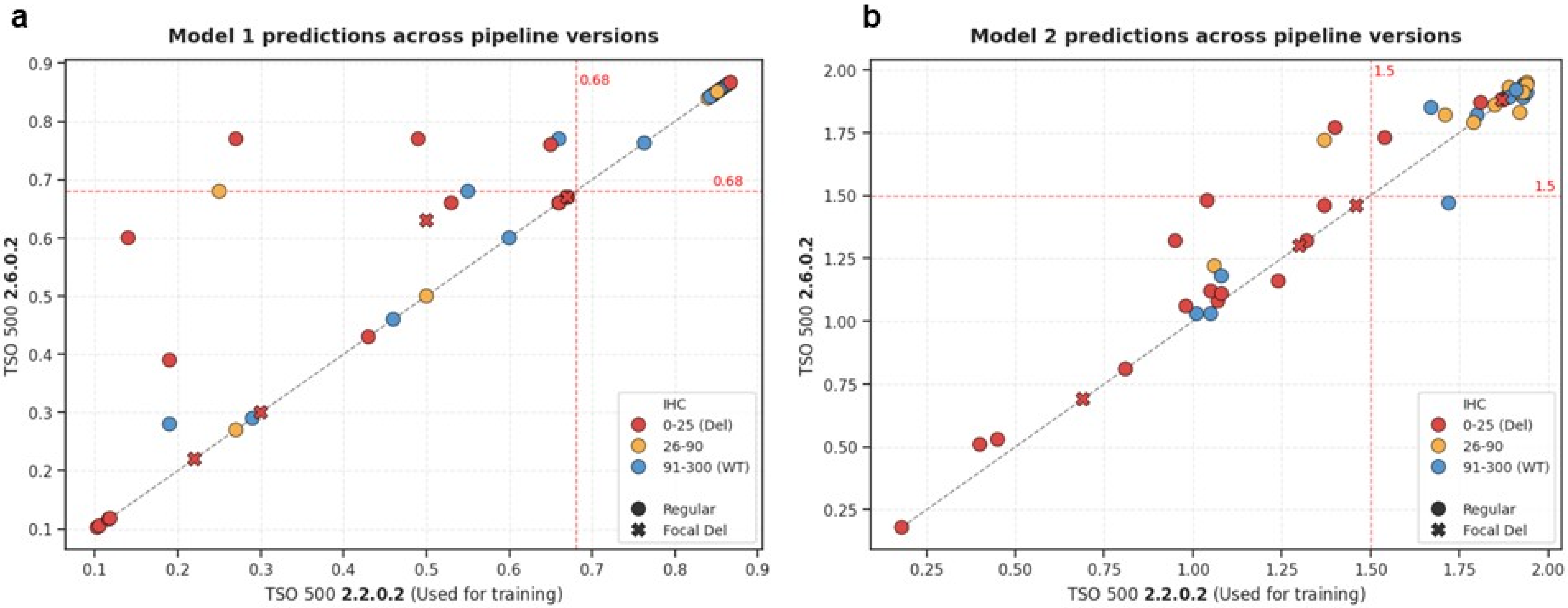

a
Model 1 predictions across pipeline versions
0.68
0.68
TSO 500 2.6.0.2
TSO 500 2.2.0.2 (Used for training)
IHC
0-25 (Del)
26-90
91-300 (WT)
Regular
Focal Del
b
Model 2 predictions across pipeline versions
1.5
1.5
TSO 500 2.6.0.2
TSO 500 2.2.0.2 (Used for training)
IHC
0-25 (Del)
26-90
91-300 (WT)
Regular
Focal Del

Supplementary Figure 3 Performance of model prediction on the external validation cohort. TSO500 and visual bin plot analysis classification showed the lowest sensitivity (0.56), whereas Model 1 and 2 classified deletions with higher sensitivity scoring 0.67 and 0.72, respectively.

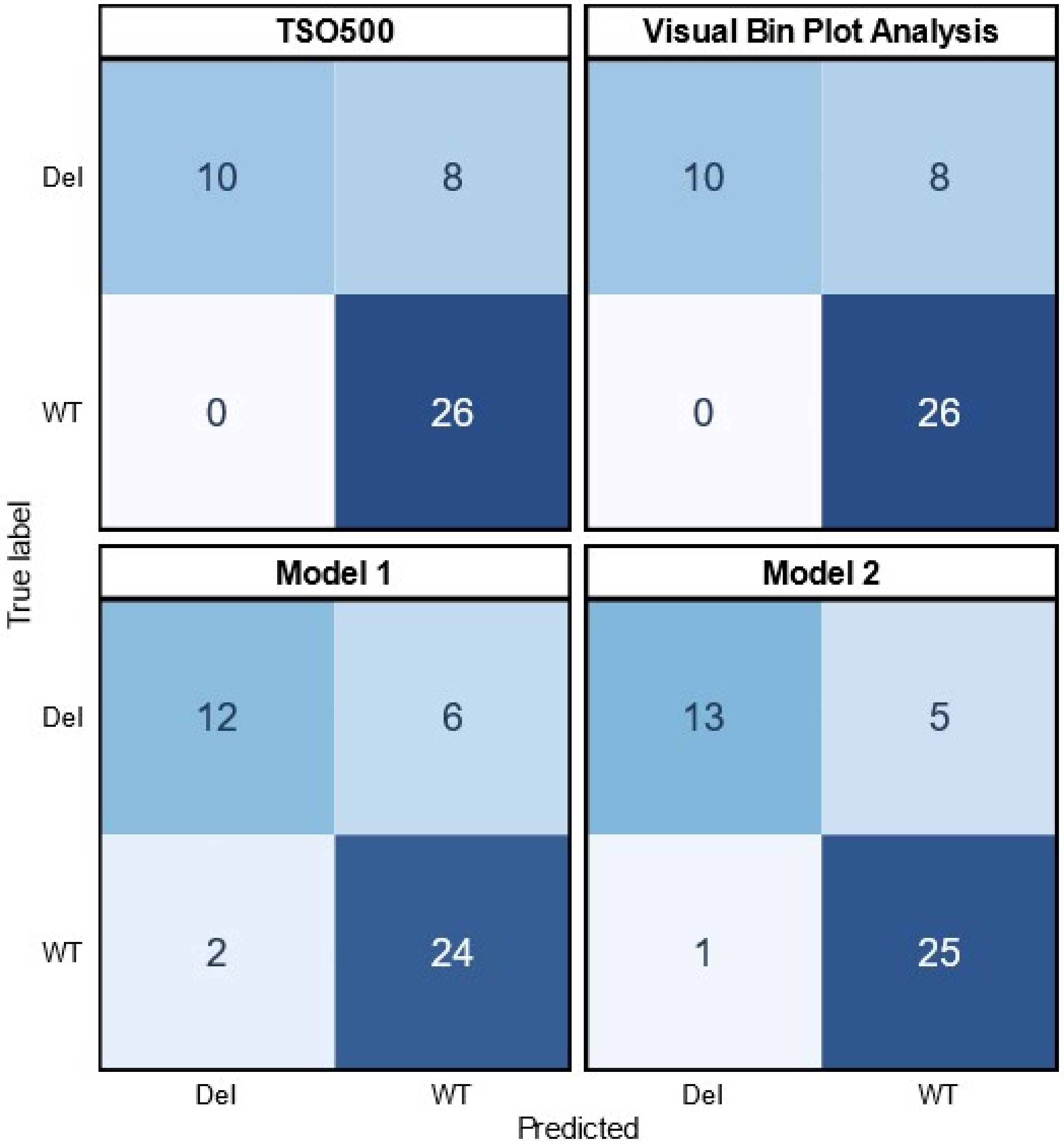
TSO500
Visual Bin Plot Analysis
Model 1
Model 2
Del
WT
10
8
0
26
10
8
0
26
12
6
2
24
13
5
1
25
True label
Predicted

Supplementary Figure 4 SHapley Additive exPlanations (SHAP) analysis of validation cohort. **a** SHAP heatmap table for PTEN bin features. Rows represent bins, columns show raw count (NormCount), and 2nd-order polynomial fit (p2_log) and its derivative (p2_der_log) for Model 1 (left) and Model 2 (right). Marked bins align with known exonic (diamond) or enhancer (triangle) regions as defined by the Ensembl project (https://projects.ensembl.org/). Darker cells indicate larger SHAP values for each model. Model 1 focuses exclusively on raw counts of six bins, whereas Model 2 distributes importance across polynomial-based features. **b** Distribution of PTEN bins in the training dataset, annotated with SHapley Additive exPlanations (SHAP) values from Models 1 and 2. Grey shaded regions highlight bins that directly influence model output, while red regions mark SHAP thresholds between positive and negative contributions. Small red and yellow bricks on the right indicate PTEN exons and enhancers, respectively, as defined by the Ensembl project.

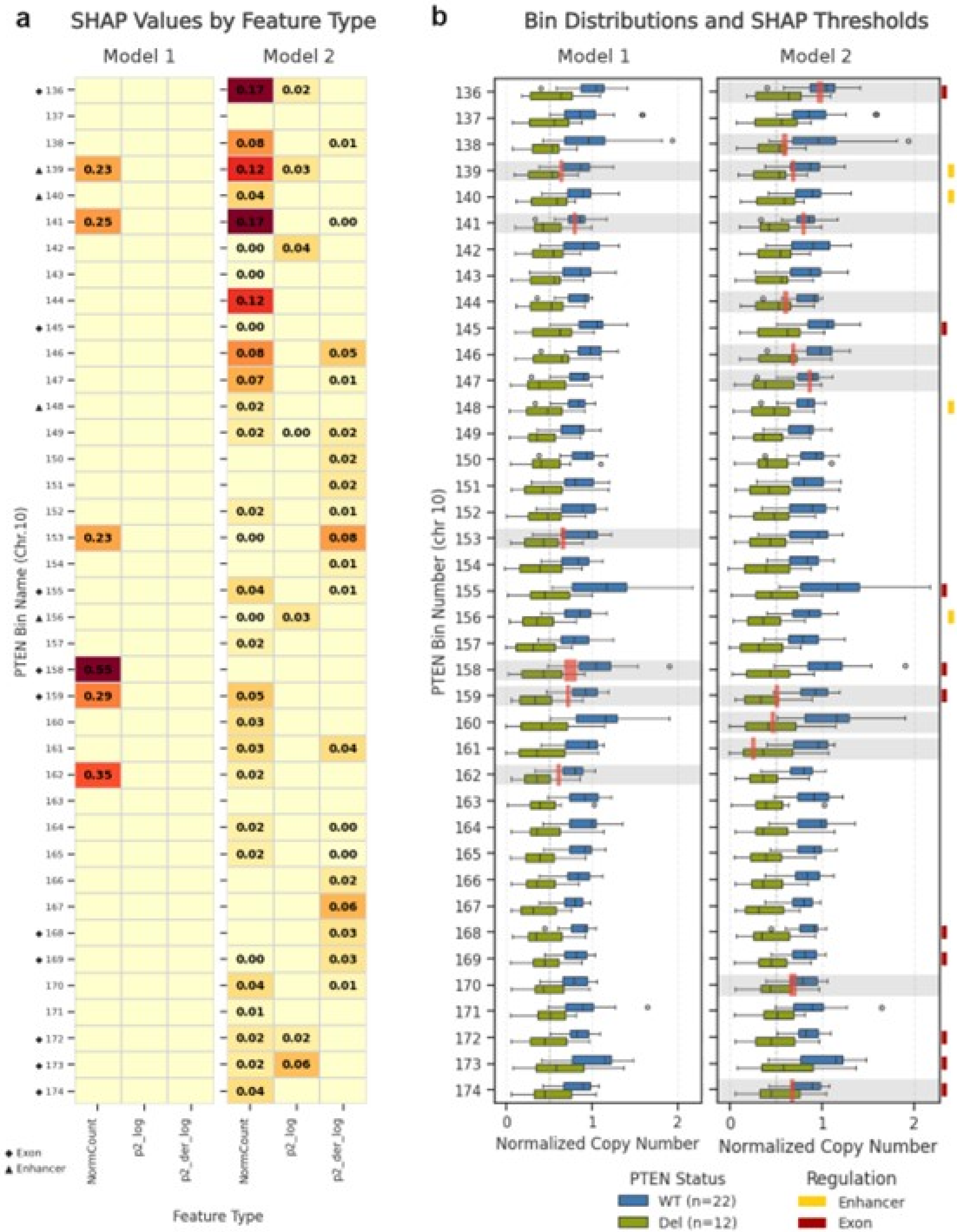
a
SHAP Values by Feature Type
Model 1
Model 2
PTEN Bin Name (Chr.10)
Feature Type
NormCount
p2_log
p2_der_log
Exon
Enhancer
b
Bin Distributions and SHAP Thresholds
Model 1
Model 2
PTEN Bin Number (chr 10)
Normalized Copy Number
PTEN Status
WT (n=22)
Del (n=12)
Regulation
Enhancer
Exon
